# SARS‐CoV‐2 mRNA Vaccination Leads to Transient Humoral and B Cell Bystander Responses in Adults

**DOI:** 10.1002/eji.70127

**Published:** 2026-01-07

**Authors:** Lisan H. Kuijper, Laura Y. L. Kummer, Laura Fernandez Blanco, Karlijn van der Straten, Mathieu A. F. Claireaux, Amélie V. Bos, Miranda C. Dieker‐Meijer, Tineke Jorritsma, Mariël C. Duurland, Maurice Steenhuis, Juan J. Garcia Vallejo, Koos P. J. van Dam, Eileen W. Stalman, Luuk Wieske, Sander W. Tas, Laura Boekel, Gert‐Jan Wolbink, Theo Rispens, Taco W. Kuijpers, Filip Eftimov, Marit J. van Gils, Anja ten Brinke, S. Marieke van Ham, T2B! immunity against SARS‐CoV‐2 study group

**Affiliations:** ^1^ Sanquin Research and Landsteiner Laboratory of the Academic Medical Center University of Amsterdam Amsterdam the Netherlands; ^2^ Amsterdam Institute for Immunology and Infectious Diseases Amsterdam the Netherlands; ^3^ Department of Neurology and Neurophysiology Amsterdam UMC Amsterdam the Netherlands; ^4^ Department of Medical Microbiology and Infection Prevention Amsterdam UMC Amsterdam the Netherlands; ^5^ Department of Molecular Cell Biology and Immunology Amsterdam UMC Amsterdam the Netherlands; ^6^ Department of Clinical Neurophysiology St. Antonius Hospital Nieuwegein the Netherlands; ^7^ Amsterdam Rheumatology and Immunology Center Amsterdam UMC Amsterdam the Netherlands; ^8^ Amsterdam Rheumatology and Immunology Center Reade Amsterdam the Netherlands; ^9^ Department of Pediatric Immunology, Rheumatology and Infectious Disease Amsterdam UMC Amsterdam the Netherlands; ^10^ Swammerdam Institute for Life Sciences University of Amsterdam Amsterdam the Netherlands

**Keywords:** antibodies, antibody‐secreting cells, DN3, IgG+ activated B cells, memory B cells, noncognate antigen, serological memory

## Abstract

After antigen encounter, long‐lived antibody‐secreting cells (ASC) secrete high‐affinity circulating antibodies. In addition, memory B cells (MBC) are quickly reactivated upon antigen re‐exposure and predominantly generate shorter‐lived ASCs. Studies have suggested that MBC can differentiate into ASCs without recognizing their cognate antigen, a process known as “bystander activation”. This antigen‐independent reactivation of MBC could help maintain circulating antibody levels, thereby protecting against future infections. To elucidate whether SARS‐CoV‐2 mRNA vaccination leads to bystander activation of B cells, the dynamics of antibody concentrations against six pathogen‐specific antigens not encountered during the sampling period were analyzed over time. Deep profiling of antigen‐specific B cell responses was simultaneously performed using multiparameter high‐dimensional spectral flow cytometry. Antibody concentrations against tetanus toxoid (TT), respiratory syncytial virus (RSV), and influenza hemagglutinin (HA) unexpectedly increased 6 weeks after the first SARS‐CoV‐2 vaccination. Deep profiling of B cell differentiation stages demonstrated a short‐term increase in influenza‐specific IgG+ DN3 B cells, RSV‐specific IgG+ CD11c+ activated B cells, and TT‐specific IgG+ MBC following vaccination. In this study, we demonstrated at both the antibody and cellular levels that SARS‐CoV‐2 mRNA vaccination transiently activates distinct early activated B cell compartments directed against influenza HA, RSV, and TT.

## Graphical abstract

SARS‐CoV‐2 mRNA vaccination triggers bystander activation of B cells, transiently increasing antibody levels after the first vaccination against influenza, RSV, and tetanus. Reactivation of B cell responses also occurred, where (CD11c^+^) MBC and DN3 are expanded after SARS‐CoV‐2 mRNA vaccination, independent of antigen exposure.

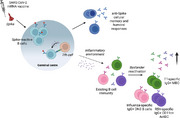

**Abbreviations**
ASC: antibody‐secreting cell
GC: germinal center
HA: hemagglutinin
MBC: memory B cell
NBC: naïve B cell
RSV: respiratory syncytial virus
Tfh: T follicular helper cells
TT: tetanus toxoid

## 1 Introduction

Class‐switched, high‐affinity antibody responses induced upon infection or vaccination play an important role in protection against many pathogens. Long‐lived antibody responses are produced and maintained by bone‐marrow resident long‐lived antibody‐secreting cells (ASC). Additionally, memory B cells (MBC) formed during the initial response can quickly reactivate and differentiate into short‐lived ASCs upon antigen re‐exposure. Typically, B cell differentiation is initiated by antigen‐specific B cell receptor (BCR) stimulation, followed by additional differentiation signals from follicular T helper cells (Tfh). Studies have demonstrated antigen‐independent polyclonal MBC activation upon influenza HA or TT vaccination, or following infection with measles virus or varicella zoster virus [1, 2, 3, 4, 5]. Since long‐lived ASCs may not be maintained indefinitely, it has been hypothesized that circulating antibody levels can be sustained by recurrent non‐BCR‐triggered MBC differentiation or homeostatic MBC differentiation into mainly short‐lived ASCs [2, 4, 6, 7, 8] and possibly even a more long‐lived ASC fraction [2]. This process has been coined “bystander activation”.

In contrast to naïve B cells (NBC), switched MBCs can be activated by stimuli other than BCR recognition [2]. Toll‐like receptor (TLR) triggering and/or cytokine stimulation, such as IL‐4 from noncognate Tfh cells [9, 10], can be sufficient to induce MBC proliferation and differentiation into an ASC phenotype [2, 7, 8]. Moreover, IgG+ switched MBCs appear to have a lower activation threshold and higher sensitivity to noncognate T cell activation compared with IgM+ MBCs [2, 11, 12]. Together, these data suggest that MBCs can respond in two ways: an antigen‐dependent manner through BCR‐triggering by cognate antigen, and a noncognate BCR‐independent process, by responding to environmental stimuli such as TLR triggering and cytokines. Data on bystander activation remain limited and are complicated by the actual occurrence of cross‐reactive B cell activation, which in itself would not be true bystander activation.

This study allowed us to test the hypothesis that de novo SARS‐CoV‐2 mRNA vaccination can lead to BCR‐independent bystander activation. Therefore, in the current study, we investigated both longitudinal antibody and B cell responses specific to six non‐SARS‐CoV‐2 antigens, including influenza HA, RSV, and TT, following SARS‐CoV‐2 mRNA vaccination.

## 2 Results and Discussion

### 2.1 SARS‐CoV‐2 Vaccination Increases Humoral Responses against Tetanus Toxoid, RSV‐F, and Influenza HA

**FIGURE 1:**
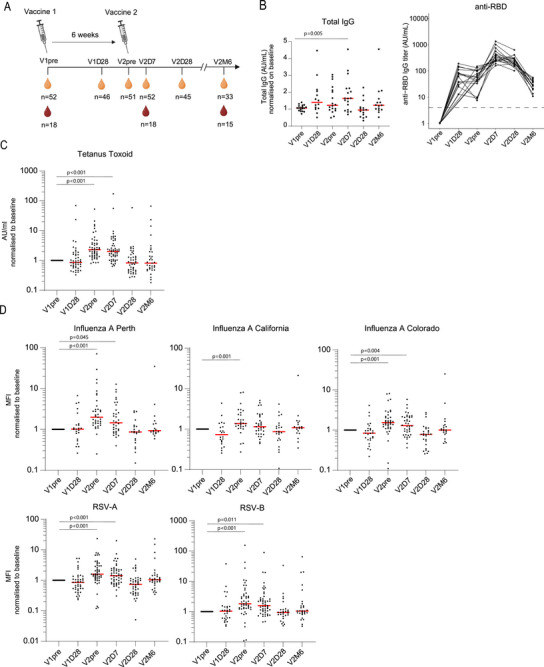
Increase in humoral immune response to noncognate antigen vaccination. (A) Study design with timing of SARS‐CoV‐2 mRNA‐1273 vaccination and peripheral blood mononuclear cell (PBMC, in red) and serum (in yellow) collection. (B) Total IgG concentration and RBD‐IgG measured at different timepoints after SARS‐CoV‐2 mRNA vaccination. (C) Normalized IgG concentrations against Tetanus Toxoid measured using ELISA at different timepoints. (D) Normalized IgG concentrations against Influenza A strains Perth, California, and Colorado, and RSV‐A and RSV‐B, measured using the Luminex assay at different timepoints. Concentrations for C–D were normalized to V1pre, and statistical analysis was performed by comparing all timepoints to V1pre. Statistical significance was assessed using the Wilcoxon signed‐rank test for paired data, and *p*‐values were corrected for multiple comparisons using post hoc Bonferroni–Holm's test. Nonsignificant values are not shown.

To investigate whether SARS‐CoV‐2 mRNA vaccination (mRNA‐1273) promotes nonspecific B cell activation, antibody levels against other antigens were analyzed. IgG antibodies specific to TT, RSV type A and B fusion proteins (RSV‐A and RSV‐B), and three influenza HA strains were measured in 52 COVID‐19 naïve non‐immunocompromised adults. Due to the lockdown during the sample collection period, exposure to RSV or influenza was unlikely. Furthermore, participants did not receive TT or influenza vaccinations during the study period. Serological responses were measured before the first vaccination (V1pre), 28 days after the first vaccination (V1D28), 6 weeks after the first vaccination at the timepoint before the second vaccination (V2pre), and seven days, 28 days, and 6 months after second vaccination (V2D7, V2D28, V2M6) (Figure 1A). Total IgG concentrations increased slightly at V1D28 and significantly at V2D7 compared with baseline (Figure 1B). These peaks correspond to the increase in anti‐RBD IgG titres at these timepoints following vaccination with SARS‐CoV‐2 (Figure 1B). Interestingly, we observed a significant increase in TT‐specific IgG antibodies at V2pre and V2D7 post SARS‐CoV‐2 vaccination (Figure 1C,E1A). This increase in TT‐specific antibodies then declined back to levels before vaccination at timepoint V2D28 (Figure 1C,E1A). To confirm this finding for other antigen specificities, serum IgG concentrations against three Influenza HA and two RSV strains were measured using a Luminex assay (Figure 1D,E1B). Indeed, IgG concentrations specific to Influenza‐A‐Perth, Influenza‐A‐California, and Influenza‐B‐Colorado were significantly increased 6 weeks postvaccination (Figure 1D,E1B). Influenza‐A‐Perth and Influenza‐B‐Colorado specific concentrations were still elevated at the 7 weeks after vaccination timepoint (V2D7). RSV‐A and RSV‐B IgG concentrations were also significantly expanded 6 and 7 weeks after vaccination (Figure 1D,E1B). Together, these data show that SARS‐CoV‐2 mRNA vaccination increases non‐SARS‐CoV‐2 circulating IgG antibody levels against distinctly different pathogens. Furthermore, noncognate IgG concentrations behaved dynamically differently from vaccine‐specific IgG response, as noncognate antibody levels were highly transient and peaked at week 6–7 after the first vaccination.

### 2.2 Shifts in Noncognate Antigen‐Specific B Cell Phenotype Following SARS‐CoV‐2 Vaccination

**FIGURE 2:**
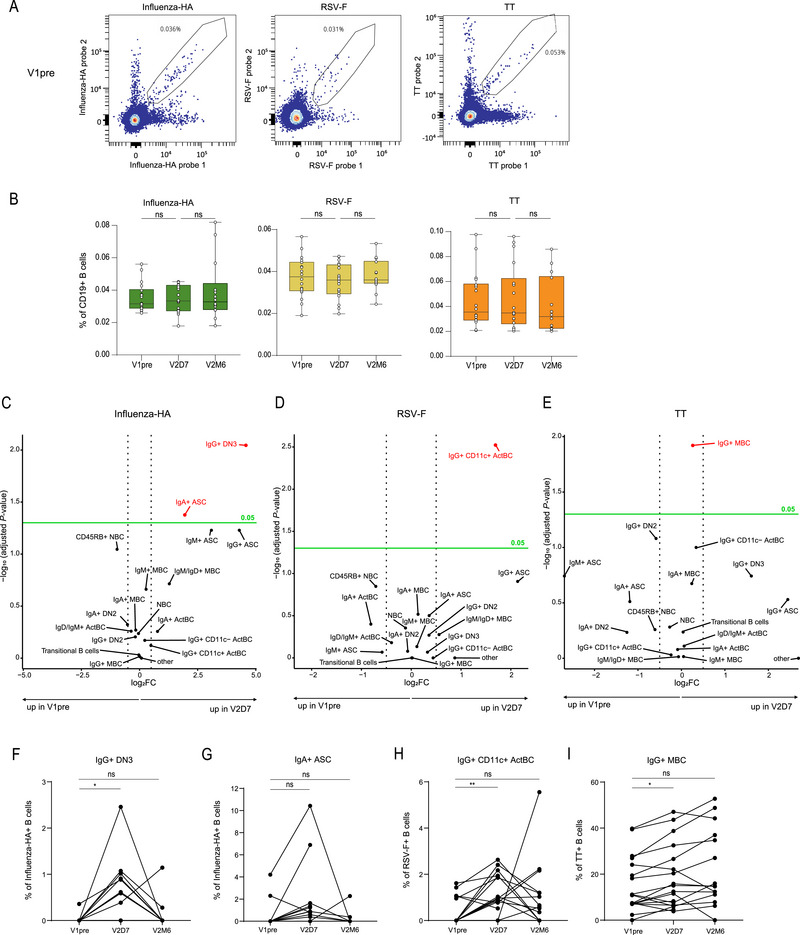
Early activated B cell clusters increased after noncognate antigen vaccination. (A) Example of a gating strategy for antigen‐specific B cell acquisition. Detection of antigen‐specific B cells using dual‐probe labelling to detect double‐positive Influenza HA‐, RSV‐F‐ or TT‐specific cells. (B) Frequency of Influenza‐, RSV‐ or TT‐specific B cells in total CD19+ B cells at three timepoints after vaccination. (C–E) Volcano plot depicting mean differences between V1pre and V2D7 for antigen‐specific B cell populations for (C) Influenza‐HA, (D) RSV‐F, and (E) TT. The fold change difference between ag‐specific B cell populations is shown on the *x*‐axis, populations with positive FC are higher expressed in V2D7, and with negative FC are higher in V1pre. The dotted line indicates the threshold set for fold change, at −0.5 and 0.5, respectively. Statistical difference is shown on the y‐axis, with a solid green line indicating a 0.05 *p*‐value, with populations above this line (therefore statistically different) shown in red. (F–I) Statistically different populations from (C–E) are illustrated here. Antigen‐specificity is shown on the y‐axis. Comparison of different timepoints (V1pre and V2D7, and V2D7 and V2M6) is further highlighted. Statistical significance was assessed using the Wilcoxon signed‐rank test for paired data, and *p*‐values were corrected for multiple comparisons using post hoc Bonferroni–Holm's test. Nonsignificant values are not shown (*p < 0.05, **p < 0.01, ***p < 0.0005).

Based on the increase in TT, RSV, and influenza antibody responses observed following SARS‐CoV‐2 vaccination, we next evaluated whether we could observe phenotypic changes in the noncognate B cells. PBMC samples were available from a select number of donors (Figure 1A). Circulating antigen‐specific B cells were investigated using dual fluorescently‐labelled multimerized recombinant proteins, including Influenza‐HA‐2009 (H1) protein, RSV‐A Fusion protein (F), and TT. B cell responses were analyzed at timepoints measured in this study (V1pre, V2D7, and V2M6) (Figure 2A). To specifically study B‐cell responses for potential bystander effects, we measured the dynamics of B cells specific to a single antigen, ensuring no cross‐reactivity with other antigens. Influenza‐HA, RSV‐F, and TT‐specific B cells were detected in all participants, and frequencies of total antigen‐specific B cells remained constant over time following SARS‐CoV‐2 vaccination (Figure 2B). To investigate whether the differentiation stages of these non‐SARS‐CoV‐2‐specific B cells changed upon SARS‐CoV‐2 vaccination, deep‐phenotyping of the antigen‐specific B cell compartment was performed using unsupervised clustering (Figure E2) [13, 14]. FlowSOM clustering was performed on antigen‐specific CD19+ B cells (HA, RSV‐F, TT, and SARS‐CoV‐2 Spike), using a selection of core lineage markers (Figure E2). Clustering analysis revealed 16 overarching B cell populations that could be divided into 38 clusters [13]. Briefly, we found several MBC (CD27+CD71−) and ASC (CD27+CD38+) populations, along with double negative 2 (DN2; IgD−CD27−CD11c+) and double negative 3 (DN3; IgD−CD27−CD11c−) populations, which are associated with the extrafollicular response [15, 16]. Additionally, we identified IgG+ activated B cells (ActBCs; CD27+CD21−CD71+), which have been described as precursors for long‐lived MBC [17, 18]. For this study, we split the IgG+ ActBC population into a CD11c+ and a CD11c− cluster, as these clusters show different contraction dynamics following vaccination [13].

For each antigen (influenza HA, RSV‐F, and TT), the frequency of antigen‐specific B cell clusters was compared between V1pre and V2D7 (Figure 2C–E). Thresholds for significant *p*‐values and fold change were set to identify clusters that were significantly differentially expressed. At V2D7, influenza‐HA‐specific IgG+ DN3 cells significantly increased compared with baseline (Figure 2C). Additionally, influenza‐HA IgA+ ASC also significantly increased at V2D7, and IgG+ and IgM+ ASC showed a trend for expansion at this timepoint (Figure 2C). Furthermore, RSV‐F‐specific IgG+ CD11c+ ActBCs increased at V2D7, which was not observed for IgG+ CD11c− ActBCs (Figure 2D). This aligns with finding of Horns and colleagues, who demonstrated that reactivated MBCs can gain CD11c+ expression following influenza vaccination [3]. Lastly, TT‐specific IgG+ MBC frequencies significantly increased at V2D7 compared with baseline, although the change was minimal (Figure 2E). In summary, while the total noncognate B cell populations remained stable over time, changes in the phenotypic distribution of early activated B cell clusters were observed in Influenza‐HA, RSV‐F, and TT‐specific B cells following SARS‐CoV‐2 mRNA vaccination.

To investigate whether the changes in influenza HA, RSV‐F, and TT‐specific B cell phenotypes were transient, we determined the frequency of these populations at V2M6 (Figure 2F–I). Influenza‐specific IgG+ DN3, RSV‐specific IgG+ CD11c+ ActBCs, and TT‐specific IgG+ MBCs significantly increased at V2D7 compared with V1pre and returned to baseline levels at V2M6 (Figure 2F,H,I), indicating a transient response. Notably, the significant increase (*p* = 0.042) in influenza HA‐specific IgA+ ASCs observed between V1pre and V2D7 was lost when correcting for multiple comparisons (*p* = 0.084) (Figure 2G). In summary, changes in noncognate antigen‐specific B cell phenotypes following SARS‐CoV‐2 vaccination were temporary.

## 3 Concluding Remarks

In conclusion, our findings demonstrate at both the serological and cellular levels that SARS‐CoV‐2 mRNA vaccination promotes bystander activation of noncognate B cells. SARS‐CoV‐2 mRNA vaccination leads to a short‐lived boost of noncognate antibody levels. Increased IgG concentrations were observed for TT, Influenza‐A‐Perth, Influenza‐A‐California, Influenza‐B‐Colorado, and RSV‐A and RSV‐B. This response was observed 6 weeks after the first vaccination, and although the increase was still present at V2D7 for most antibody levels, this was likely still an effect of the first immune activation. No additional changes in noncognate antibody levels were detected after the second vaccination; however, samples 6 weeks after the second vaccination were not available.

This study was mainly constrained by limited timepoints for cellular analysis. Cellular samples were available for only a select number of donors, and no cellular sampling was performed after the first vaccination and 6 weeks after the second vaccination. Therefore, it remains uncertain whether the phenotypic changes in the Influenza HA, RSV F, and TT‐specific B cells observed at V2D7 result from ongoing responses after the first vaccination or derive directly from the second vaccination. IgG+ CD11c+ ActBC show hallmarks of extrafollicular activation, as opposed to IgG+ CD11c− ActBC. DN3 has also been implicated to play a role in the extrafollicular response, despite lacking CD11c expression [15]. This suggests that DN3 and CD11c+ ActBC could arise from MBC reactivation, as no antigen for TT, RSV, or Influenza was present for de novo immune cell formation. A study looking into chronic malaria exposure showed that TT‐specific B cells with an atypical memory B cell phenotype were increased compared with nonchronic malaria exposure, suggesting a chronic inflammatory environment can also change noncognate B cell phenotype [19].

Possibly, the stimulatory environment created in response to vaccination, such as the induction of a GC response, the release of cytokines, Tfh activation, and innate immune responses, may have caused bystander B cell activation. An alternative explanation may lie in the fact that SARS‐CoV‐2 mRNA vaccines consist of single‐stranded RNA, which serves two roles: encoding the viral spike protein and inducing innate immune activation via TLR stimulation, thus serving as an adjuvant. Single‐stranded RNA can stimulate TLR7 and induce a strong type 1 interferon response directly via B cells or indirectly via dendritic cells, enhancing T and B cell expansion [20]. B cells established after both infection (Influenza and RSV) as well as vaccination (TT) appear to respond after SARS‐CoV‐2 vaccination. Altogether, this study provides evidence for bystander activation of noncognate B cells following vaccination for a different antigen (Supporting Information).

## Author Contributions

A.T.B. and S.M.V.H. supervised the study. F.E., T.W.K., S.M.V.H., and A.T.B. conceptualised the study. A.T.B., L.F.B., A.B., L.H.K., N.J.M.V., T.R., M.C., J.J.G‐V, M.J.V.G., and M.S. designed the methodology. L.H.K., L.Y.L.K., L.F.B., A.B., M.C.D., T.J., M.D.M., K.V.D.S. performed the experiments. L.H.K., L.Y.L.K., L.F.B., K.V.D.S, M.J.V.G, S.M.V.H, and A.T.B. verified the overall replication/reproducibility of the research output. L.H.K., L.Y.L.K., and L.F.B. analyzed the data. L.Y.L.K., E.W.S., L.B., G.W., and S.W.T. provided study materials. L.H.K., L.Y.L.K., L.W., and E.W.S. managed the research data. L.H.K., L.Y.L.K., L.W., E.W.S., T.R., T.W.K, F.E., S.M.V.H., and A.T.B. managed and coordinated the research activity and planning. T.W.K., F.E., and S.M.V.H. acquired the financial support for the project leading to this publication. L.H.K. and L.Y.L.K. designed and implemented computer codes. L.H.K., L.F.B., and L.Y.L.K. visualised the work. L.H.K., L.Y.L.K., and L.F.B. wrote the original draft. All authors reviewed and approved the manuscript.

## Funding

This research project was supported by ZonMw (The Netherlands Organization for Health Research and Development, #10430072010007). L.H.K. and M.C.D. are funded by the Sanquin Blood Supply program grant PPOC OPTIMAL, project number L2506.

## Conflicts of Interest

Filip Eftimov, Gert‐Jan Wolbink, S. Marieke van Ham, and Taco W. Kuijpers report (governmental) grants from ZonMw to study immune response after SARS‐CoV‐2 vaccination in autoimmune diseases. Filip Eftimov also reports grants from Prinses Beatrix Spierfonds, CSL Behring, Kedrion, Terumo BCT, Grifols, Takeda Pharmaceutical, and GBS‐CIDP Foundation; consulting fees from UCB Pharma and CSl Behring; and honoraria from Grifols. All other authors report no conflicts of interest.

## Collaborators

T2B! Immunity against SARS‐CoV‐2 study group: Renée CF van Allaart, Adája E Baars, Marcel W Bekkenk, Frederike J Bemelman, Angela L Bosma, Bo Broens, Esther Brusse, Matthias H Busch, Olvi Cristianawati, Pieter A van Doorn, George Elias, Cécile ACM van Els, Marit J van Gils, H Stephan Goedee, Geert RAM D'Haens, Dirk Jan Hijnen, Marc L Hilhorst, Barbara Horváth, Papay BP Jallah, Mark Löwenberg, Elham S Mirfazeli, Annelie H Musters, Jim BD Keijser, Sofie Keijzer, Joep Killestein, Karina de Leeuw, Anneke J van der Kooi, Lotte van Ouwerkerk, Pieter van Paassen, Agner R Parra Sanchez, W Ludo van der Pol, Nicoline F Post, Joost Raaphorst, Annabel M Ruiter, Abraham Rutgers, Corine RG Schreurs, Phyllis I Spuls, R Bart Takkenberg, YK Onno Teng, Yosta Vegting, Jan JGM Verschuuren, Adriaan G Volkers, Alexandre E Voskuyl, Jelle de Wit, Diane van der Woude and Koos AH Zwinderman.

## Supporting information




**Supporting File 1**: eji70127‐sup‐0001‐SuppMat.pdf.

## Data Availability

Data supporting the findings of this study are available from the corresponding author upon reasonable request.
