# Supplementary material for: SARS‐CoV‐2 mRNA Vaccination Leads to Transient Humoral and B Cell Bystander Responses in Adults
Source: Eur J Immunol. 2026 Jan 7;56(1):e70127. doi: 10.1002/eji.70127 (PMC12779779; doi:10.1002/eji.70127)
Supplement: Supplementary file 1 — Supporting File 1: eji70127‐sup‐0001‐SuppMat.pdf. [file EJI-56-e70127-s001.pdf]

Figure E1

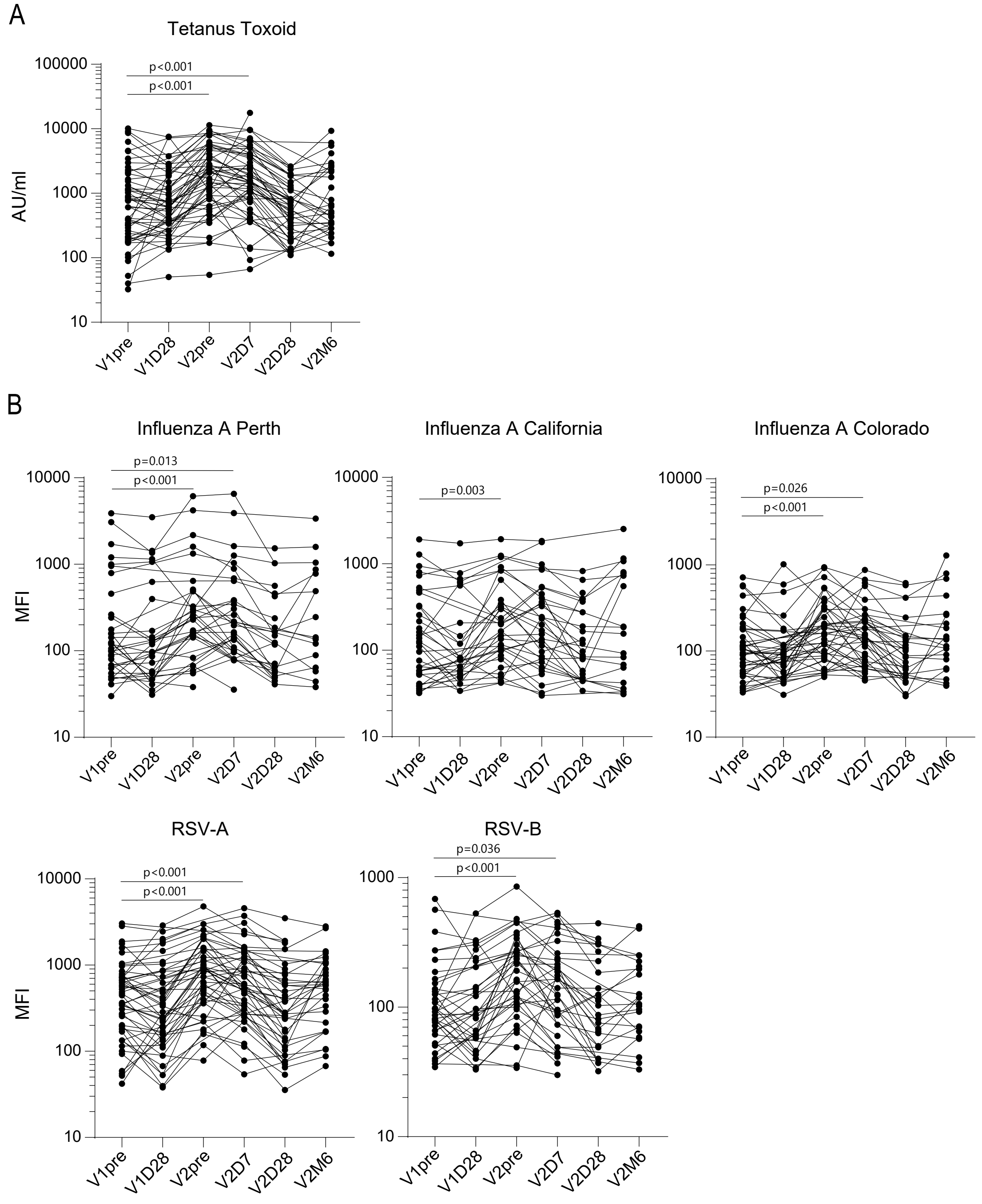
A
Tetanus Toxoid
p<0.001
p<0.001
AU/ml
100000
10000
1000
100
10
V1pre
V1D28
V2pre
V2D7
V2D28
V2M6
B
Influenza A Perth
p=0.013
p<0.001
Influenza A California
p=0.003
Influenza A Colorado
p=0.026
p<0.001
MFI
10000
1000
100
10
RSV-A
p<0.001
p<0.001
RSV-B
p=0.036
p<0.001
V1pre
V1D28
V2pre
V2D7
V2D28
V2M6

Figure E1: Humoral immune response to Tetanus Toxoid, RSV and Influenza

A) Non-normalized IgG concentrations (AU/ml) against Tetanus Toxoid measured using ELISA at different timepoints. B) Non-normalized IgG depicted as MFI against Influenza A strains Perth, California and Colorado, and RSV-A and -B measured using Luminex assay at different timepoints. Statistical analysis for A-B was performed by comparing all timepoints to V1pre. Statistical significance was assessed using Wilcoxon signed-rank test for paired data and p-values were corrected for multiple comparison using post hoc Bonferroni-Holm's test. Non-significant values are not shown.

Figure E2

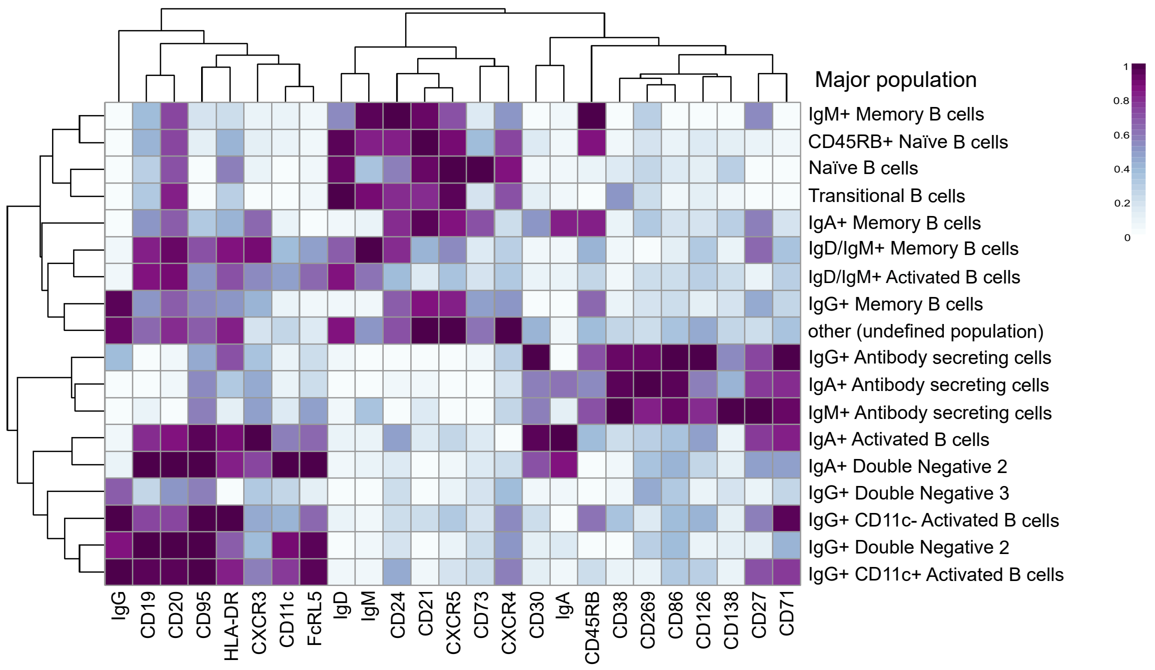
Major population
IgM+ Memory B cells
CD45RB+ Naïve B cells
Naïve B cells
Transitional B cells
IgA+ Memory B cells
IgD/IgM+ Memory B cells
IgD/IgM+ Activated B cells
IgG+ Memory B cells
other (undefined population)
IgG+ Antibody secreting cells
IgA+ Antibody secreting cells
IgM+ Antibody secreting cells
IgA+ Activated B cells
IgA+ Double Negative 2
IgG+ Double Negative 3
IgG+ CD11c- Activated B cells
IgG+ Double Negative 2
IgG+ CD11c+ Activated B cells
IgG
CD19
CD20
CD95
HLA-DR
CXCR3
CD11c
FcRL5
IgD
IgM
CD24
CD21
CXCR5
CD73
CXCR4
CD30
IgA
CD45RB
CD38
CD269
CD86
CD126
CD138
CD27
CD71
1
0.8
0.6
0.4
0.2
0

Figure E2: Identification of antigen-specific B cells populations

Antigen-specific B cells were detected using dual-probe labelling and stained with surface markers shown on bottom of the heatmap (x-axis). Different B cell populations were annotated using expression of surface markers that defined certain populations. Shown is the relative protein marker expression for each annotated B cell population (right on heatmap, y-axis).

Methods

Study design and participants

This study is part of a prospective observational cohort study in the Netherlands, called the Target-to-B! SARS-CoV-2 vaccination study including healthy individuals and patients with autoimmune disease. Full study details have been previously published(21). The study was approved by the medical ethical committee (NL74974.018.20 and EudraCT 2021-001102-30, local METC number: 2020_194) and registered at the Dutch Trial register (trial ID: NL8900). All participants provided written informed consent before participation. Participants were vaccinated between April 2021 and October 2021 with the mRNA-1273 (Moderna) SARS-CoV-2 vaccine. Two vaccinations were provided with a six week interval conform the Dutch national vaccination campaign guidelines at the time. Peripheral blood was collected by venipuncture seven days and six months post second vaccination. Peripheral blood mononuclear cells (PBMCs) were isolated within 12 hours and frozen in liquid nitrogen until further use. In total 104 participants of the Target-to-B! study cohort were used for the analysis of antigen-specific B cells. In the current study we focused the analysis on the healthy controls. Exclusion criteria were active or previous autoimmune-, oncological-, or haematological disease; current or previous treatment with systemic immunosuppressive therapy the past year, pregnancy and previous SARS-CoV-2 infection (as evidenced by positive anti-RBD antibodies before first vaccination and/or self-reported positive PCR and/or positive anti-nucleocapsid protein (NCP) antibodies throughout the study period).

Tetanus toxoid ELISA

To detect total IgG antibodies directed against TT, an ELISA was performed. A 96-well plate was coated with 100 µL of 0.5ug/mL Tetanus Toxoid (Purified TT, 150 Lf/mL, lot 6014079, gift from Netherlands Vaccine Institute Bilthoven) in PBS for three hours at 37°C and washed five times with PBS + 0.02% v/v Tween-20 (PBS-T; Merck, Germany). Serum samples diluted 1:2000 in PBS + 0.1% v/v Tween-20 + 2 g/L gelatin (PTG; Merck, Germany) were added to coated wells and incubated for 1 hour on a shaker at RT. After washing five times with PBS-T, 100 µL of 0.25ug/mL IgG-HRP antibody (MH16-1-HRP, Sanquin, ref M9084) in PTG was added to the wells and samples were incubated for 0.5

hours on a shaker at RT. After washing, 100 µL of Ultra tetramethylbenzidine substrate (TMB; Thermo Scientific) diluted 1:1 in milli-Q was added. After 10 min incubation at RT the reaction was stopped with 100 µL 0.2M H2SO4. Optical density (OD) was read at 450-540 nm with a Synergy 2 microplate reader (BioTek Instruments). TT read-outs were compared to a human normal immunoglobulin solution (Nanogam, 100 mg/mL, MD2012.02 EUDRACT: 2012-001960-31) standard curve (3-fold diluted, 8 dilutions starting at 200 µg/mL).

Luminex assays

Luminex assay was performed to test serum IgG antibody levels against RSV-F and Influenza HA, as previously described(22). In short, the RSV-F-A , RSV-F-B, Influenza-A-Perth (H3N2: A/Perth/16/2009; 40043-V08H), Influenza-A-California (H1N1: A/California/04/2009, 11055-V08H) and Influenza-B-Colorado (B/Vic: B/Colorado/06/2017, 40581-V08H) protein were covalently coupled to Luminex Magplex beads (Luminex) using a two-step carbodiimide reaction, as previously described(22). Following protein coupling to Luminex beads, 50 µGL of serum (diluted 100.000-fold) was mixed with 50 µL of bead-protein mixture (20 beads/µL) and incubated overnight at 4°C. Plates were washed the following day with TBS 0.05% Tween-20 (TBST), resuspended in 50 µL goat-anti-human IgG-PE (Soutern Biotech) and incubated at room temperature for 2 hours. Beads were washed and resuspend in 70 µL Magpix drive fluid (Luminex). Plates were read-out using Magpix (Luminex). Antibody binding was measured as Median Fluorescence Intensity (MFI) of 50 to 100 beads per well, adjusted for background signals by subtracting the MFI of wells containing only buffer and beads, and then converted into binding antibody units per ml (BAU/ml) according to the WHO International Standard for anti-SARS-CoV-2 immunoglobulin (NIBSC 20/136). Positive and negative controls were included on each plate and 36% of samples were remeasured to confirm assay results. Results with MFI<30 were excluded as these fell within the range of background detection.

Antigen-specific B cell detection

Antigen probe design, purification, and antigen-specific B cell staining were performed as previously described(13,14,23) Briefly, PBMCs were thawed in IMDM (Lonza) containing 10% FCS (Bodinco BV). Next, 10 x 10^6 PBMCs were depleted of CD3+ cells using

EasySep™ Human CD3 Positive Selection Kit II (StemCell Technologies) as per manufacturer's instructions. To stain antigen-specific B cells, biotinylated protein antigens influenza hemagglutinin (HA; H1N1pdm2009)(24), prefusion stabilized glycoprotein from respiratory syncytial virus (RSV; DS-Cav1)(25) and Tetanus Toxoid (TT; Vcar-Lsx003, Creative Biolabs) were individually multimerized with fluorochrome-conjugated streptavidin in a 2:1 molar ratio at 4°C for 1 hour on a shaker. Then, 10% D-biotin (GeneCopoeia) was added to each multimerized protein antigen and incubated at 4°C for 30 minutes to minimalize cross-reactivity. All biotinylated antigens were conjugated to two fluorochromes. A 31-color spectral cytometry panel, including the six biotinylated antigens, was designed to immunophenotype antigen-specific B cells(13). Thawed PBMC samples were first stained with Live/Dead Fixable Blue Stain Kit (Invitrogen) in PBS for 30 min at 4°C. Cells were washed with staining buffer containing 1% BSA and 1 mM EDTA in phosphate-buffered saline, and then stained with the above mentioned spectral cytometry panel, including the protein antigens, for 30 min at 4°C. Cells were washed with staining buffer, fixed with cold paraformaldehyde 1% for 10 min at RT, and washed again twice with staining buffer. Data were acquired on Cytek Aurora 5L using SpectroFlo® software (Cytek Biosciences). Briefly, FlowSOM clustering was performed using a selection of core markers (CD20, CD21, CD27, CD138, CD38, CD24, CD45RB, CD11c, IgM, IgA, IgG, IgD) on all SARS-CoV-2 (S, RBD) and other (TT, RSV-F, HA) antigen-specific CD19+ B cells. Clustering analysis revealed 16 overarching B cell populations that could be divided in 38 clusters. A more detailed description of the analysis pipeline has been described previously (13,14). We identified two CD11c+ clusters and four CD11c- clusters in the IgG+ ActBC compartment, which led us to subdivide these into IgG+ CD11c+ population and IgG+ CD11c- ActBC respectively.

Statistical analysis

All statistical analyses were performed using Rstudio (version 4.1.1). Statistical significance was determined using the Wilcoxon rank sum test for unpaired comparisons and the Wilcoxon signed-rank test for paired data. Bonferroni-Holm's method of multiple comparisons was used to correct p values in case of multiple testing. P values lower than 0.05 were considered statistically significant.

21. Wieske L, van Dam KPJ, Steenhuis M, Stalman EW, Kummer LYL, van Kempen ZLE, et al. Humoral responses after second and third SARS-CoV-2 vaccination in patients with immune-mediated inflammatory disorders on immunosuppressants: a cohort study. Lancet Rheumatol. 2022;4(5):e338–50.

22. Grobben M, van der Straten K, Brouwer PJ, Brinkkemper M, Maisonnasse P, Dereuddre-Bosquet N, et al. Cross-reactive antibodies after SARS-CoV-2 infection and vaccination. Elife. 2021;10(e70330).

23. Claireaux M, Elias G, Kerster G, Kuijper LH, Duurland MC, Paul AGA, et al. Deep profiling of B cells responding to various pathogens uncovers compartments in IgG memory B cell and antibody-secreting lineages. Sci Adv. 2025;11(8):eado1331.

24. Aartse A, Eggink D, Claireaux M, van Leeuwen S, Mooij P, Bogers WM, et al. Influenza a virus hemagglutinin trimer, head and stem proteins identify and quantify different hemagglutinin-specific b cell subsets in humans. Vaccines (Basel). 2021;9(7):717.

25. McLellan JS, Chen M, Joyce MG, Sastry M, Stewart-Jones GBE, Yang Y, et al. Structure-Based Design of a Fusion Glycoprotein Vaccine for Respiratory Syncytial Virus. Science (1979). 2013;342(6158):592–8.
